# Genetic variants in transforming growth factor-β gene *(TGFB1*) affect susceptibility to schizophrenia

**DOI:** 10.1007/s11033-013-2662-8

**Published:** 2013-09-25

**Authors:** Dorota Frydecka, Blazej Misiak, Jan Aleksander Beszlej, Lidia Karabon, Edyta Pawlak-Adamska, Anna Tomkiewicz, Anna Partyka, Anna Jonkisz, Andrzej Kiejna

**Affiliations:** 1 Department of Psychiatry, Wroclaw Medical University, Pasteura 10, 50-367 Wroclaw, Poland; 2 Department of Experimental Therapy, Laboratory of Immunopathology, Institute of Immunology and Experimental Therapy, Polish Academy of Sciences, Weigla 12, 51-114 Wroclaw, Poland; 3 Department of Forensic Medicine, Wroclaw Medical University, Mikulicza-Radeckiego 4, 50-368 Wroclaw, Poland; 4 Department and Clinic of Urology, Wroclaw Medical University, Borowska 213, 50-556 Wroclaw, Poland

**Keywords:** Schizophrenia, Interleukin, Transforming growth factor, Genetic polymorphism, Cytokine, Interferon

## Abstract

Immense body of evidence indicates that dysfunction of immune system is implicated in the etiology of schizophrenia. The immune theory of schizophrenia is supported by alterations in cytokine profile in the brain and peripheral blood. Given the strong genetic background of schizophrenia, it might be assumed that aberrant production of cytokines might be the consequence of genetic factors. This study aimed at investigating the association between schizophrenia susceptibility and selected functional polymorphisms in genes encoding cytokines including: interleukin-2 (*IL2* −330T>G, rs2069756), interleukin-6 (*IL*-*6* −174G>C, rs1800795), interferon-γ (*IFNG* +874T>A, rs2430561) as well as for the first time transforming growth factor-β1 (*TGFB1* +869T>C, rs1800470 and +916G>C, rs1800471). We recruited 151 subjects with schizophrenia and 279 controls. There was a significant difference in the genotype distribution and allelic frequency of the *TGFB1* +869T>C between patients with schizophrenia and healthy controls (*p* < 0.05). The risk of schizophrenia was more than two-fold higher in carriers of T allele (CT+TT genotypes) than individuals with CC genotype. Given documented gender differences in incidence of schizophrenia, we conducted separate analyses of male and female participants. We have shown that the association was significant in females, while in males it reached a trend toward statistical significance. To the best of our knowledge, it is the first report showing the association between *TGFB1* +869T>C polymorphism and schizophrenia.

## Introduction

Schizophrenia is a severe mental illness that affects around 0.5 % of the general population [1]. Most researchers agree that the onset of schizophrenia in women appears later than in men [2]. This finding indicates that some gender differences occur in schizophrenia and make its etiology more complex and multidimensional. There is a considerable number of theories including i.e. aberrant immune response that have been proposed in order to explain the etiology of schizophrenia. Dysfunction of immune system in schizophrenia is accompanied by altered expression of various cytokines both in the brain and peripheral blood. Indeed, schizophrenia is associated with the imbalance in Th1>Th2 cytokines towards a relative predominance of Th2 cytokines [3]. This imbalance is mainly observed in three distinct lines of cytokine profile alterations: decreased serum level of IL-2 and increased production of IL-6, as well as reduced mitogen-stimulated IFN-γ due to immune system overstimulation [4]. IL-2 stimulates growth, differentiation and proliferation of lymphocytes, particularly Th1 cells and NK cells [5]. In turn, IL-6 is a pleiotropic Th2 cytokine, produced by lymphocytes, macrophages, monocytes, as well as astrocytes and microglia [6]. Most interestingly, it stimulates the synthesis of catecholamines [7, 8]. In addition, overproduction of IL-6 has been found to correlate with clinical indices of schizophrenia [9, 10] and antipsychotic treatment decreases the plasma level of IL-6 [6]. The next cytokine, IFN-γ which is a Th1 cytokine plays a key role in antiviral defense, stimulates macrophage activity and induces expression of major histocompatibility complex (MHC) antigens [11, 12]. Deregulation of cytokine expression occurs along with elevated TGF-β1 production, which is a Th3 cytokine and has been found to suppress the production of Th1 cytokines [13].

Although dysfunction of immune response in schizophrenia has been repeatedly reported and meta-analyzed [14, 15], the primary causative factor of this phenomenon remains unknown. Given the strong hereditary background of schizophrenia, it is feasible to assume that genetic factors may also underlie immune system deregulation and aberrant cytokine production observed in schizophrenia. Therefore, several authors have focused on functional polymorphisms located in genes encoding cytokines implicated in schizophrenia. However, data in respect to particular genes, especially *IL2, INFG* and *IL6* are limited and inconsistent, therefore do not allow to draw unequivocal conclusions. In this study, we aimed to investigate the association between following polymorphisms: *IL2* −330T>G (rs2069756), *IL6* −174G>C (rs1800795), *INFG* +874T>A (rs2430561) in the Polish population and for the first time to investigate the role of selected *TGFB1* polymorphisms—*TGF*-*β1* +869T>C (rs1800470) and *TGF*-*β1* +913G>C (rs1800471) in susceptibility to schizophrenia.

## Materials and methods

### Subjects

We recruited 151 patients with schizophrenia (89 females and 69 males of mean age 38.0 ± 11.9) and 279 controls (61 females and 196 males of mean age 38.7 ± 8.8). All subjects were of Caucasian origin and they were entirely native, unrelated Polish population recruited from the same geographic area—Lower Silesia region. The study was approved by Institutional Ethics Committee and all participants provided written informed consent prior to participation.

All the patients were recruited from Wroclaw Medical University Hospital. They had a confirmed diagnosis of schizophrenia by the same two senior board-certified investigators according to DSM-IV criteria (Diagnostic and Statistical Manual of Mental Disorders, Fourth Edition, 1994) based on individual interviews, clinical observation, medical records (hospital and outpatient clinic case notes) and family information. Additionally, all patients were evaluated for lifetime psychotic symptomatology using the Operational Criteria for Psychotic Illness (OPCRIT) checklist, which provides a polydiagnostic categorical and dimensional approach to a diagnosis of schizophrenia [16]. The OPCRIT provides a convenient, reliable, rapid, and valid approach to psychiatric assessment that can be used as an alternative to the conventional best-estimate consensus diagnostic procedures. It has been used in a wide variety of clinical, epidemiological, and biological research applications. The patients having a history of traumatic brain injury, neurologic disorders, substance addiction (with exception of nicotine) were excluded from the study by a detailed medical examination. The control group consisted of healthy volunteers free from present, past and family history (first-degree relatives) of psychiatric illness and the exclusion criteria for the patients.

### Genotyping

Genomic DNA was obtained from peripheral white blood cells from whole frozen blood using the QIAamp DNA Blood Mini Kit (Qiagen GmbH, Hilden, Germany). IL2 −330T>G (rs2069756) polymorphism was examined using allelic Real Time PCR discrimination method with TaqMan SNP Genotyping Assay—C__15859930_10. The following polymorphisms *IL6* −*174G*>*C* (rs1800795)*, IFNG* +*874T*>*A* (rs2430561)*, TGFB1* +*869T*>*C* (rs1800470) and *TGFB1* +*913G*>*C* (rs1800471) were examined by the PCR with sequence specific primers technique (PCR-SSP) using the PCYTGEN kit (One Lambda, Canoga Park, USA). PCR products have been visualized on 2 % agarose gel.

### Statistical analyses

Evaluation of the Hardy–Weinberg equilibrium (HWE) was performed by comparing the observed and expected genotype distribution using the χ^2^ test. The χ^2^ analysis was also used to compare categorical data between patients with a diagnosis of schizophrenia and healthy controls subjects. In case when observed or expected values included a cell with a value <5, Fisher’s exact test was used. Odds ratios (OR) and 95 % confidence intervals (95 % CI) were calculated using the binary logistic regression model. Differences were considered as statistically significant if the *p* value was <0.05. Bonferroni adjustments were applied to the level of significance due to the multiple comparisons in *TGFB1* gene. Analyses were performed using SHEsis software platform [17].

## Results

All results for patients and controls were in HWE. Allele frequency and genotype distribution for each tested polymorphism are presented in the Table 1. The OR and 95 % CI are shown for the codominant model. We found that allele frequency and genotype distribution of the *TGFB1* +869T>C polymorphism differed significantly between schizophrenia patients and healthy controls (*p* = 0.03 and 0.02 respectively) (Table 1). Application of Bonferroni correction revealed a significant difference with regard to genotype distribution (*p*_after Bonfferoni correction_ = 0.04), while only a trend-level significance with respect to difference in allele frequency (*p*_after Bonfferoni correction_ = 0.06). Notably, the T carriers (CT+TT genotypes) were significantly more frequent in schizophrenia group then in healthy controls (89 vs. 78 %, *p* = 0.006, *p*_after Bonfferoni correction_ = 0.012, OR 2.22, 95 %CI 1.24–3.95). The frequency of alleles and genotypes for the other investigated cytokine gene polymorphisms did not differ significantly between schizophrenia patients and control subjects (Table 1).

**Table 1:** Genotype distribution and allele frequencies of polymorphisms in *IL2*, *INFG*, *IL6* and *TGFB1* genes in schizophrenia patients and healthy controls

Polymorphism	Schizophrenia patients (%)	Controls (%)	OR	95 % CI	*p* value*
*IL2* −330T>G (rs2069756)					
TT	64 (43.0)	129 (47.0)	Referent	Referent	0.72
TG	69 (46.3)	118 (43.1)	1.18	0.77–1.80	
GG	16 (10.7)	27 (9.9)	1.19	0.60–2.37	
T allele	197 (66.1)	376 (68.6)	Referent	Referent	0.46
G allele	101 (33.9)	172 (31.4)	0.89	0.66–1.20	
*IL6* −174G>C (rs1800795)					
GG	40 (26.5)	82 (29.9)	Referent	Referent	0.72
GC	72 (47.7)	128 (46.7)	1.15	0.72–1.85	
CC	39 (25.8)	64 (23.4)	1.25	0.72–2.16	
G allele	152 (50.3)	292 (53.3)	Referent	Referent	0.41
C allele	150 (49.7)	256 (46.7)	1.13	0.85–1.49	
*IFNG* +874T>A (rs2430561)					
AA	50 (33.6)	74 (27.1)	Referent	Referent	0.38
TA	74 (49.7)	147 (53.8)	1.34	0.85–2.12	
TT	25 (16.8)	52 (19.1)	1.40	0.77–2.55	
T allele	124 (41.6)	251 (46.0)	Referent	Referent	0.22
A allele	174 (58.4)	295 (54.0)	1.94	0.89–1.59	
*TGFB1* +869T>C (rs1800470)					
CC	17 (11.3)	61 (21.9)	Referent	Referent	**0.02**
TC	78 (51.6)	127 (45.7)	2.20	1.20–4.04	**0.04** ^**†**^
TT	56 (37.1)	90 (32.4)	2.23	1.18–4.20	
C allele	112 (37.1)	249 (44.8)	Referent	Referent	**0.03**
T allele	190 (62.9)	307 (55.2)	1.38	1.03–1.83	
*TGFB1* +913G>C (rs1800471)					
GG	124 (82.1)	232 (84.4)	Referent	Referent	0.74
GC	25 (16.6)	41 (14.9)	1.14	0.66–1.96	
CC	2 (1.3)	2 (0.7)	1.87	0.26–13.44	
G allele	273 (90.4)	502 (91.9)	Referent	Referent	0.48
C allele	29 (9.6)	44 (8.1)	1.19	0.73–1.95	

* *p* value calculated with the χ^2^ test except for the *TGFB1* +913G>C polymorphism (*p* value calculated in the Fisher exact test)

^**†**^ *p* value after application of Bonferroni correction

Bold value indicates that *p* value is less than 0.05 (*p* < 0.05)

Additionally, we conducted separate analyses for male and female participants. Allele frequency and genotype distribution of each tested polymorphism with respect to gender are presented in the Table 2. We found that the difference in genotype distribution and allele frequency of the TGFB +869T>C polymorphism was significant between females with a diagnosis of schizophrenia and healthy controls (*p* = 0.018, *p*_after Bonfferoni correction_ = 0.036 and *p* = 0.008, *p*_after Bonfferoni correction_ = 0.016 respectively), but not between male participants (Table 2). In women suffering from schizophrenia the presence of T allele (CT+TT genotypes) was observed more frequently than in healthy women (87 vs. 69 %, *p* = 0.006, *p*_after Bonfferoni correction_ = 0.012, OR 2.86, 95 %CI 1.33–6.18), while in men with a diagnosis of schizophrenia there was only a trend toward overrepresentation of T allele observed (91.9 vs. 81 %, *p* = 0.043, *p*_after Bonfferoni correction_ = 0.086, OR 2.67, 95 %CI 1.0–7.14). Allele frequency and genotype distribution of the other investigated cytokine gene polymorphisms with regard to gender did not differ significantly between schizophrenia patients and healthy controls (Table 2).

**Table 2:** Genotype distribution and allele frequencies of polymorphisms in *IL2*, *INFG*, *IL6* and *TGFB1* genes in schizophrenia patients and healthy controls with regard to gender

Polymorphism	Males		OR	95 % CI	*p* value*	Females		OR	95 % CI	*p* value*
	Schizophrenia patients (%)	Controls (%)				Schizophrenia patients (%)	Controls (%)			
*IL2* −330T>G (rs2069756)										
TT	29 (47.5)	87 (44.85)	Referent	Referent	0.87	34 (39.1)	42 (52.5)	Referent	Referent	0.21
TG	27 (44.3)	87 (44.85)	0.93	0.51–1.96		42 (48.3)	31 (38.8)	1.67	0.88–3.20	
GG	5 (8.2)	20 (10.3)	0.23	0.07–0.70		11 (12.6)	7 (8.7)	1.94	0.68–5.55	
T allele	85 (69.7)	261 (67.3)	1.12	0.72–1.74	0.62	110 (63.2)	115 (71.9)	Referent	Referent	0.09
G allele	37 (30.3)	127 (32.7)	Referent	Referent		64 (36.8)	45 (28.1)	0.67	0.42–1.07	
*IL6* −174G>C (rs1800795)										
GG	14 (22.6)	64 (33.0)	Referent	Referent	0.18	26 (29.2)	18 (22.6)	Referent	Referent	0.28
GC	35 (56.4)	85 (43.8)	1.88	0.93–3.79		37 (41.6)	43 (53.7)	0.57	0.28–1.25	
CC	13 (21.0)	45 (23.2)	1.32	0.57–3.08		26 (29.2)	19 (23.7)	0.95	0.40–2.20	
G allele	63 (50.8)	213 (54.9)	Referent	Referent	0.43	89 (50.0)	79 (49.4)	Referent	Referent	0.91
C allele	61 (49.2)	175 (45.1)	1.18	0.79–1.77		89 (50.0)	81 (50.6)	0.97	0.64–1.49	
*IFNG* +874T>A (rs2430561)										
TT	10 (16.4)	32 (17.1)	Referent	Referent	0.73	15 (17.0)	16 (20.3)	Referent	Referent	0.29
TA	38 (62.3)	107 (57.2)	1.14	0.51–2.53		36 (40.9)	39 (49.4)	0.98	0.42–2.27	
AA	13 (21.3)	48 (25.7)	0.87	0.34–2.21		37 (42.0)	24 (30.3)	1.64	0.69–3.93	
T allele	58 (47.5)	171 (45.7)	Referent	Referent	0.75	66 (37.5)	71 (44.9)	Referent	Referent	0.17
A allele	64 (52.5)	203 (54.3)	0.93	0.62–1.40		110 (62.5)	87 (55.1)	1.36	0.88–2.11	
*TGFB1* +869T>C (rs1800470)										
CC	5 (8.1)	37 (19.0)	Referent	Referent	0.12	12 (13.5)	25 (30.9)	Referent	Referent	
TC	33 (53.2)	88 (45.1)	2.77	1.00–7.66		45 (50.6)	36 (44.4)	2.62	1.15–5.89	**0.018**
TT	24 (38.7)	70 (35.9)	2.26	0.79–6.46		32 (36.1)	20 (24.7)	3.33	1.37–8.09	**0.036** ^**†**^
C allele	46 (37.7)	162 (41.5)	Referent	Referent	0.17	69 (38.8)	86 (53.1)	Referent	Referent	**0.008**
T allele	76 (62.3)	228 (58.5)	1.17	0.77–1.78		109 (61.2)	76 (46.9)	1.79	1.16–2.75	**0.016** ^**†**^
*TGFB1* +913G>C (rs1800471)										
GG	52 (83.9)	169 (86.7)	Referent	Referent	0.83	72 (80.9)	63 (78.8)	Referent	Referent	0.56
GC	9 (14.5)	24 (12.3)	1.22	0.53–2.79		16 (18.0)	17 (21.2)	0.82	0.38–1.76	
CC	1 (1.6)	2 (1.0)	1.62	0.14–18.3		1 (1.1)	0 (0)	–	–	
G allele	113 (91.1)	362 (92.8)	Referent	Referent	0.54	160 (89.9)	143 (89.4)	Referent	Referent	0.88
C allele	11 (8.9)	28 (7.2)	1.26	0.61–2.61		18 (10.1)	17 (10.6)	0.95	0.47–1.91	

* *p* value calculated in the χ^2^ test except for the *TGFB1* +913G>C polymorphism (*p* value calculated with the Fisher exact test)

^**†**^ *p* value after application of Bonferroni correction

Bold value indicates that *p* value is less than 0.05 (*p* < 0.05)

## Discussion

To the best of our knowledge, it is the first study examining the role of two common polymorphisms located in *TGFB1* gene in schizophrenia. We found that the presence of *TGFB1* +869T>C [T] allele increased susceptibility to disease about two fold. *TGFB1* gene is located within the chromosome 5q31–32, the region that has been most consistently associated with schizophrenia in genome-wide linkage analyses [18]. Moreover, in the recent association study of 5q31–32 region, DNA pooling analysis revealed that *TGFB1* gene is also a candidate gene for schizophrenia [19]. In the combined analysis of genome-wide association studies (GWAS) from Genetic Association Information Network (GAIN) [20], TGF-β signaling pathway was one of the top ranked pathways associated with schizophrenia [21]. Although the *TGFB1* +869T>C polymorphism (rs1800470) was not associated with schizophrenia, another polymorphism in this gene (rs2241714) reached significant *p* value (0.003). The functional study indicated that the presence of the *TGFB* +869T>C [T] allele correlates with increased production of TGF-β [22].

These findings are in agreement with the immune theory of schizophrenia. One of the most consistent findings with regards to the immune deregulation in schizophrenia is the imbalance between Th1 and Th2 lymphocytes towards the predominance of Th2 cells [3, 23–25]. Since TGF-β1 inhibits the immune response of Th1, it might facilitate the imbalance between two major subpopulations of lymphocytes [26]. Additionally, in the recent meta-analysis of cytokine alterations it has been shown that the plasma level of TGF-β1 is significantly increased during exacerbations of schizophrenia [14]. Our findings suggest that this alteration may occur due to genetic variation in *TGFB1* gene. For many years now, the multifunctional TGF-β signaling machinery has been described in the central nervous system. It has been shown that TGF-β signaling is a crucial factor controlling neural stem cells maintenance and differentiation, determining growth and size of the developing brain [27]. Additionally, in vitro and in vivo evidence shows that TGF-β receptors are expressed in naïve neurites during embryonic development, playing role in axon differentiation and neuronal migration in the developing neocortex [28]. In animal models, TGF-β1 promotes survival of midbrain dopaminergic neurons [29] and mediates dopamine inhibition of pituitary lactotrophs activity [30]. Moreover, TGF-β expression is induced following a variety of types of brain tissue injury, exerting neuroprotective functions and attenuating brain damage through anti-inflammatory, -apoptotic, -excitotoxic actions as well as through the angiogenesis and neuroregeneration promotion [31].

Moreover, we found that genotype distribution and allele frequency of the *TGFB1* +869T>C polymorphism is gender-specific. The *TGFB1* +869T>C [T] allele was significantly more frequent in schizophrenia females than in healthy women, whereas this association did not reach statistical significance in males. In this regard, we provided additional evidence supporting the role of gender differences in the etiology of schizophrenia. Gender differences occur in several aspects of schizophrenia including epidemiological indices, age of onset, premorbid functioning, course and outcome of the disorder, subtle neurodevelopmental brain malformations and treatment response [2]. These findings suggest that hormonal and sexual dimorphism is implicated in the etiological dilemma of schizophrenia. Overwhelming evidence suggests that estradiol is the key contributor of gender-specific alterations that are observed in schizophrenia. Notably, estradiol interacts with various neurotransmission systems and it has been shown that low plasma estradiol level accompanies exacerbations of schizophrenia in women [32]. In the light of our results, it is of great importance that TGF-β and estradiol interact in a feedback loop and the course of action of this feedback seems to be tissue specific. For instance, estradiol has been found to induce the release and expression of TGF-β in cortical and hypothalamic astrocytes [33, 34] as well as in the pituitary [30], whereas in peripheral tissues e.g. in dermal fibroblasts may inhibit the production of TGF-β [35]. Similarly, the effect of TGF-β on the production of estradiol remains also complex. Although it has been found that TGF-β promotes estrogen production via enhancing the basal secretion of follicle-stimulating hormone (FSH) [36], in vitro studies have provided contradictory results with regard to the influence of TGF-β on ovarian cells [37]. Therefore, future studies should determine the reciprocal interactions between estrogens and TGF-β in the brain regions that are affected in schizophrenia. It should also be mentioned that gender differences are sparsely taken into consideration in studies looking into association of cytokine genes polymorphisms with schizophrenia. Thus, some important connections with regard to gender dimorphism in schizophrenia might have been overlooked so far. Our data along with the measurement of plasma estrogens level warrant the need for further studies that would strengthen the link between TGF-β1 signaling and gender differences in schizophrenia.

Notably, we were unable to find a significant association between the remaining polymorphisms (*IL2* −330T>G, *IL6* −174G>C, *IFNG* +874T>A, *TGFB1* +913G>C) and schizophrenia in our study.

*IL2* −330T>G polymorphism is located within the binding domains for transcription factors in the promoter region of *IL2* gene. The study on peripheral blood lymphocytes stimulated by anti-CD3>CD28 antibodies revealed that the *IL*-*2* −330T>G [GG] genotype contributes to increase in the production of IL-2 while the *IL*-*2* −330T>G [GT] and the *IL*-*2* −330T>G [TT] genotypes are associated with reduced production of IL-2 [38]. The studies with respect to *IL2* −330T>G polymorphism in schizophrenia are limited and reporting conflicting results. In the study performed by Schwartz et al. [39], the *IL*-*2* −330T>G [G] allele was significantly more frequent in schizophrenia subjects than in healthy controls; however, their results did not follow HWE creating potential bias arising from population stratification. Of note, in our study we have noticed a trend for overrepresentation of G carriers in schizophrenia woman in comparison with healthy women. Contradictory results were obtained by Watanabe et al. [40], who did not confirm the association between this polymorphism and schizophrenia.

Similar inconsistencies have been reported with regard to the *IL6* −174G>C polymorphism. This polymorphism lies in the promoter region that is responsible for transcription induced by viruses, second messengers and other cytokines [41]. In the study by Fishman et al. [42], who used a luciferase reporter construct transfecting HeLa cells, a 0.624-fold decrease in expression from the *IL*-*6* −174G>C [C] allele was detected. In turn, stimulation with lipopolysaccharide and IL-1β resulted in a non-significant change of expression from the *IL*-*6* −174G>C [C] allele and a significant increase of expression from the *IL*-*6* −174G>C [G] allele (2.35- and 3.60-fold, respectively). Interestingly, the *IL6* −174G>C polymorphism does not act individually and its influence on transcription depends upon other polymorphisms in *IL6* gene [41]. This observation along with the influence of ethnicity may serve as the explanation for discordant results in studies on the *IL6* −174G>C polymorphism in schizophrenia. Indeed, Paul-Samojedny et al. [43] found a trend toward a significant difference in allelic frequency and genotype distribution of this polymorphism between schizophrenia subjects and healthy controls. In turn, in the study from Armenian population, the *IL6* −174G>C [C] allele along with higher IL-6 plasma level were associated with schizophrenia [44].

The *IFNG* +874T>A is located in the intron 1 and the +864T allele increases expression via interactions with the NF-κB transcription factor [45]. There is only one study that examined the role of *IFNG* +874T>A polymorphism in schizophrenia [46]. The authors found that this polymorphism is associated with paranoid schizophrenia in males of Caucasian origin from Poland. Frequency of the *IFNG* +874T>A[T] allele was 48.88 %, while frequency of the A allele was 51.12 % in schizophrenia subjects. These results are similar to those obtained by our group (Table 1). However, the allele frequency in healthy controls recruited by Paul-Samojedny et al. [46] (55.36 % for the *IFNG* +874T>A[T] allele and 44.64 % for the *IFNG* +874T>A[A] allele) is highly discordant with our results (Table 1). When we assessed HWE for the results by Paul-Samojedny et al. [46], we found that the genotype distribution in schizophrenia subjects follow HWE (χ^2^ = 2.04, df = 1, *p* value = 0.15). However, the genotype distribution in healthy controls significantly deviated from HWE (χ^2^ = 70.56, df = 1, *p* value < 1 × 10^−6^). Lack of concordance with HWE may be due to the selection bias originating from population stratification, which increases the possibility of obtaining significant differences.

Discordant results of these studies might be attributed to several confounders including i.e. insufficient sample size, diversity of population characteristics, ethnic admixture and complexity of the phenotype [47]. However, scarcity of studies on selected polymorphisms points to the difficulty in drawing unequivocal conclusions.

## Conclusions

Our results indicate that the *TGFB1* +869T>C gene polymorphism is associated with schizophrenia, especially in females. This finding suggests that previously reported alterations in the plasma level of TGF-β1 may occur due to genetic variation in its gene. Furthermore, gender differences in schizophrenia might be the consequence of the interaction between female hormones and TGF-β signaling. Thus, future studies should determine the link between TGF-β and the production of estrogens in schizophrenia. Our results do not support the association of polymorphisms in *IL2*, *IL6* and *IFNG* genes with schizophrenia. Given that studies measuring the plasma level of these cytokines in schizophrenia subjects have provided relatively consistent findings that immune deregulation is implicated in schizophrenia, it might be concluded that altered production of IL-2, IL-6 and IFN-γ is not the consequence of genetic factors.
